# Intermittent fasting and neurocognitive disorders: What the evidence shows

**DOI:** 10.1016/j.jnha.2025.100480

**Published:** 2025-01-10

**Authors:** Jordan Beveridge, Allison Montgomery, George Grossberg

**Affiliations:** St. Louis University Department of Psychiatry and Behavioral Neuroscience, Monteleone Hall, 1438 South Grand Boulevard, St. Louis, MO 63104, United States

**Keywords:** Intermittent fasting, Time restricted feeding, Time restricted eating, Neurocognition, Dementia, Alzheimer’s disease, AD, TRF, IF, Geriatric, Psychiatry, Geriatric psychiatry, Lifestyle, Diet

## Abstract

**Introduction:**

Intermittent fasting (IF) has emerged as a potential lifestyle intervention for mitigating cognitive decline and enhancing brain health in individuals with mild to major neurocognitive disorders. Unlike preventive strategies, this review evaluates IF as a therapeutic approach, focusing on its effects on neuroplasticity, inflammation, and cognitive function.

**Methods:**

A narrative review was conducted using a comprehensive PubMed search with the terms “intermittent fasting AND neurocognition” and “intermittent fasting AND neuroplasticity”. Studies published in English within the last ten years involving human and animal models were included. Exclusion criteria focused on studies primarily examining mood disorders or unrelated metabolic outcomes.

**Results:**

Preclinical evidence demonstrates that IF enhances hippocampal neurogenesis and synaptic plasticity through pathways involving BDNF and CREB. IF also reduces neuroinflammation, as shown in animal models of Alzheimer’s disease, vascular cognitive impairment, and high-fat diet-induced cognitive impairment. Human studies, though limited, suggest that regular IF may improve cognitive function and reduce markers of oxidative stress and inflammation in individuals with mild cognitive impairment.

**Conclusion:**

Current findings highlight the therapeutic potential of IF for individuals with existing cognitive impairment. While preclinical studies provide robust evidence of neuroprotective mechanisms, human studies remain sparse and require standardization. Further clinical research is necessary to confirm long-term safety and efficacy and to refine IF protocols for broader clinical application.

## 1 Introduction

The health of the human brain is a major area of concern among the medical and research community and the public as we face an ageing population and increased incidence of neurocognitive disease [1,2]. Established interventions have been shown to support brain health prior to onset of neurocognitive disorder like Alzheimer’s disease (AD), not only by decreasing exposure to risk factors but through pointed lifestyle changes. Among dietary modifications, most evidence is in support of the Mediterranean diet, which is primarily plant-based with fruits, vegetables, healthy fats, and fish [3]. The Dietary Approaches to Stopping Hypertension (DASH) diet is often prescribed based on evidence showing reduction in cardiovascular risk factors, and emphasizes fruits, vegetables, and whole grains [4]. The Mediterranean-DASH Intervention for Neurodegenerative Delay (MIND) diet combines Mediterranean and DASH recommendations and is shown to reduce cognitive decline with age [5]. Evidence also supports caloric restriction (CR) in general as having positive effects on brain health, although this is both difficult to maintain long-term as well as unsuitable for individuals with preexisting low BMI or muscle mass [6,7]. However, most studies on dietary modification have been done in populations prior to development of overt neurocognitive disease. In persons affected by neurocognitive disorders, particularly AD, some studies have been enacted on interventional diets aiming to moderate cognitive decline during the disease course. For instance, there is growing evidence on the neuroprotective effects of ketone bodies [8]. A recent systematic review of ten randomized, controlled trials of the ketogenic diet (KD) in AD showed promising results. Rong et al. concluded that KD led to improved cognitive function in patients and improved brain energy metabolism through ketone use [9]. Another review concluded that both KD and exogenous ketone supplementation can lead to treatment benefit in AD patients [10]. Polyunsaturated fatty acids of the omega-3 family were shown in a longitudinal study of n = 140 to be present in plasma at higher levels in those with mild neurocognitive disorder versus patients with major neurocognitive disorder. These fatty acids are commonly found in the Mediterranean diet, possibly supporting its continued use after diagnosis of cognitive impairment [11].

Interest has grown in intermittent fasting (IF) as a potential lifestyle intervention for promoting brain health and slowing cognitive decline [12]. IF has been shown to increase levels of circulating ketones to higher levels than CR, supporting its potential for neuroprotection [13]. As more research emerges, the question is whether IF can be integrated into existing lifestyle recommendations to further support cognitive function in the face of decline. The objective of this review is to discuss IF as it relates to neuroplasticity, inflammation, and neurocognitive disorders. Rather than examining IF as a preventive strategy, this paper evaluates its potential as a therapeutic approach to mitigate existing symptoms and improve brain function in the context of early to advanced neurocognitive disorders. For these concepts, relevant mechanisms and biochemical pathways will be discussed, as well as highlighting animal studies and existing human studies. The review will conclude with implications of these findings as well as directions for future research. There are several types of intermittent fasting described in the literature, with the most common being time-restricted eating (TRE). In some cases, both IF and TRE are referred to as separate types of intermittent energy restriction (IER) [14], but for simplicity’s sake, this paper will refer to IF as the overarching term. In contrast to ad libitum (AL), in which subjects eat as much and as often as desired, IF sets time constraints for eating [15]. Time-restricted eating (TRE), or time-restricted feeding (TRF) as it is known in animal studies, involves restricting food intake to a certain number of hours per day (≤12) and fasting for the rest of the 24-h period. A common TRE profile consists of eating only during an 8-h period and fasting for the remaining 16 h [16]. Alternate day fasting (ADF) involves fasting for 24 h, then eating whenever desired for the following 24 h. Other methods of IF are described as well, and may be combined with caloric restriction, typically to 70% of normal calorie intake without malnutrition [17]. Many of the studies use AL diet as a control. The eating periods of IF diets typically allow for eating as often as desired during the allowed time interval [15,17].

Sustained intermittent fasting induces a metabolic switch from glycogenolysis to lipolysis, which generally occurs 12–36 h after initiation of the fast. This timing of this metabolic switch is influenced by factors including initial liver glycogen content, the nutritional composition of the most recent meal, and the rate of energy expenditure during the fast. The switch enables the catabolism of lipids through β-oxidation into free fatty acids and subsequently ketones, primarily β-hydroxybutyrate (BHB) and acetoacetate (AcAc). Ketones may then be used as fuel for metabolic processes [18,19]. In Alzheimer’s disease (AD), glucose metabolism is impaired, reducing the ability of the brain to utilize glucose [20]. In the context of IF, the creation of ketone bodies itself may provide an alternative fuel source for the brain and potentially allow for increased brain function in AD patients [21].

The metabolic switch involves several biochemical pathways, explaining some of the neurocognitive benefits of IF. Reduced availability of glucose and elevation of ketone bodies lower the AMP to ATP ratio in neurons, which activates kinases including Amp activated protein kinase (AMPK) and calcium calmodulin dependent protein kinase (CaKMII) as well as transcription factors including cAMP response element binding protein (CREB) and PGC1-alpha, all of which stimulate autophagy [18]. Autophagy in neurons has been shown to support neurogenesis and provide protection against stress; dysfunction of this mechanism has been seen in neurodegenerative processes [22]. Low glucose levels also decrease the activity of the mTOR growth signaling pathway, leading to autophagy. BHB itself upregulates the expression of brain-derived neurotropic factor (BDNF), which promotes mitochondrial biogenesis, synaptic plasticity, and cellular stress resistance. In addition, IF lowers circulating blood insulin levels through the insulin/IGF signaling pathway, which enhances neuroplasticity and protects against metabolic and oxidative stress [18].

## 2 Methods

A comprehensive literature search was conducted using the PubMed database to identify relevant studies exploring the relationship between intermittent fasting (IF) and neurocognitive health. The following search terms were used: “intermittent fasting AND neurocognition” and “intermittent fasting AND neuroplasticity”. The search was filtered to include articles published in English within the past ten years. Studies involving both animal and human participants were included to provide a broad understanding of the subject.

Inclusion criteria focused on studies investigating the effects of IF on neurocognitive function, mechanisms of neuroplasticity, and inflammation. Studies specifically addressing the role of IF in neurocognitive disorders, such as Alzheimer’s disease and mild cognitive impairment, were prioritized. Exclusion criteria included studies primarily examining IF’s impact on mood disorders or unrelated metabolic outcomes, per the guidance to maintain a focused review scope.

Access to full-text articles was provided through institutional affiliations with Saint Louis University (SLU). Relevant references from included studies were further reviewed to ensure comprehensive coverage of the topic and avoid omitting recent or significant findings.

This review was conducted as a narrative review and aimed to provide a broad overview of existing literature and synthesize findings without following the strict methodological framework of systematic reviews. Therefore, registration in PROSPERO or similar platforms was not applicable in this context.

## 3 Results

### 3.1 Intermittent fasting and neuroplasticity

#### 3.1.1 IF increases hippocampal neurogenesis

Neuroplasticity, or the ability of neurons to modify and strengthen synaptic connections, is an innate part of brain function and development. It is essential for learning and memory but is also studied for its ability to help rebuild after neuronal devastation such as brain injury or disease [23,24]. IF has shown potential in increasing neuroplasticity through several mechanisms, including neuronal growth in the hippocampus through the Notch and BDNF pathways. In a mouse study by Baik et al., it was demonstrated that IF vs AL diets in mice increased hippocampal neurogenesis and long-term potentiation at hippocampal synapses, strengthening the synaptic connection. The study involved four arms with mice randomized to a control of AL, fasting 12 h/day, fasting 16 h/day, or ADF (fasting 24 h every other day) and maintained on these diets for three months. Results showed that in the three IF arms, mice had higher levels of neuronal nuclei and nestin, a marker for neural stem cells, in the hippocampus compared to AL mice, indicating increased hippocampal neurogenesis. These were both significantly higher in the 16-h fasting group. In all three groups of IF mice, postsynaptic density protein-95 (PSD-95), a scaffolding protein associated with synaptic connectivity and plasticity, also increased significantly. This suggests IF plays a role in strengthening synaptic connections [25]. The Notch signaling pathway is an established pathway involved in maintenance of neural stem cells in the mammalian brain [26]. Researchers hypothesized that neurogenesis occurred via this signaling pathway and tested several known markers for Notch in the mice, including Notch 1, Notch intracellular domain, transcription factor HES5, and presenilin 1. All markers were found to be upregulated in IF groups compared to AL group after three months, with significance in the 16-h fasting group for the first three markers. Furthermore, researchers in Baik et al. found BDNF and CREB expression to be upregulated in the hippocampus of IF mice compared to AL, again resulting in a significant difference in the 16-h fasting group. As it is known that Notch collaborates with the BDNF and CREB signaling pathways involved with the differentiation of neural stem cells into neurons, the increase of both markers further suggests that IF mice had increased neuronal stem cell proliferation and differentiation leading to increased hippocampal neurogenesis [25,27].

#### 3.1.2 Role of BDNF and CREB in IF

BDNF is a nerve growth factor and an integral player in not only neuroplasticity, but also for functions including learning, memory, and mood. It is a necessity for the survival and development of neurons [28,29]. The significance of this growth factor is highlighted in studies on patients with major depressive disorder (MDD), in which BDNF is an important regulatory protein in the pathophysiology of the illness [30]. In patients with depression, plasma and serum BDNF levels are lower compared to healthy controls. A meta-analysis looking at antidepressant efficacy showed that SSRIs and SNRIs increase peripheral BDNF levels over the course of treatment compared to baseline [31]. In addition, electroconvulsive therapy (ECT), one of the most effective treatments for MDD, increased BDNF levels following therapeutic use for treatment-resistant patients with the disease [32]. BDNF and Tropomyosin receptor kinase B (TrkB) modulate neurogenesis and synaptic plasticity [33]. In MDD patients, BDNF-TrkB signaling activity is predictably more likely to be altered compared to controls- an intriguing finding given a disease with diagnostic criteria consisting only of a constellation of symptoms [31]. Deficits in BDNF signaling contribute to neurodegenerative disorders as well, raising the question of potential neuroprotective status as a target for intervention [34]. A review synthesizing 53 interventions and 29 reviews found that there is primary evidence for cognitive and neuroprotective benefits of intermittent fasting through the BDNF signaling pathway. Long-term potentiation is facilitated by self-amplification of BDNF by transcription factor CREB, a feedback cycle that is strongly implicated in maturation and proper function of synapses. While human studies in this realm at this time focus primarily on risk factors associated with chronic health conditions including metabolic syndrome and inflammation, animal model studies show a link between IF and the improvement of cognitive performance via BDNF. In this review, BDNF and CREB were consistently found to be upregulated in the hippocampus of animals treated with IF compared to AL eating [33]. On the other hand, aging has previously been associated with a length-dependent loss of BDNF and, as expected, memory, learning, and neuroplasticity [35]. Therefore, it is logical to posit that the upregulation of BDNF through IF could contribute to mitigation of these functional losses with age.

### 3.2 Intermittent fasting and inflammation

#### 3.2.1 The role of inflammation in neurocognitive disorders

Aging is associated with changes in gene expression that increase the inflammatory and stress responses while decreasing protein turnover and growth and trophic factors [36,37]. Calorie restriction, on the other hand, directly counteracts many of these processes through decreasing the stress response and increased growth/trophic factors, DNA synthesis, and immune modulation which suppresses inflammatory genes. Through decreased oxidative stress and inflammation, calorie restriction provides a mechanism for protection against the pro-inflammatory state and neurocognitive disorders linked to aging [38]. Markers of inflammation are found in tissue associated with cognitive disorders such as AD and Parkinson Disease, and inflammatory factors are considered a potential drug target by researchers studying these conditions [39]. For instance, beta-amyloid plaques of late-stage AD have been found to have pro-inflammatory cytokines, activated microglia, and other markers. Findings such as these, as well as the association of decreased inflammation with a decrease incidence of these diseases, support the widely accepted theory that neuroinflammation leads to a decline in neurocognition [37,40]. Several studies of IF suggest that it may have a role to play in reducing neuroinflammation through various pathways, further proposing IF as a strategy for modulation of neurocognitive disease.

#### 3.2.2 Effects of intermittent fasting on neuroinflammation

A rise in neuroinflammation has been shown to be directly associated with worse prognosis after intracerebral hemorrhage (ICH), a significant brain injury studied for its prevalence in human populations and replicated in mouse models [41,42]. In Dai et al., intermittent fasting was found to reduce inflammation in hemorrhagic stroke-induced mice post-injury. Mice were randomly assigned to a non-fasting group or IF group, fed on alternate days. ICH was modeled in both groups by intrastriatal injection of autologous blood. In IF mice, morphologic results showed both acute and chronic differences: in the first week, IF-treated mice showed enhanced hematoma clearance and significantly reduced brain edema; after 28 days there was attenuated striatum atrophy in this group as well. IF mice, compared to AL mice, had more neurons preserved in the ipsilateral hemisphere both immediately and after 28 days. Additionally, IF was associated with decreased apoptosis, suppressed microglia activation, and reduced inflammatory markers IL-1 β and TNF-α. The observed changes in intermittent fasting mice were shown to be mediated through the sirtuin 3 (Sir3) pathway, which maintains mitochondrial homeostasis under stress, after Sir3 knockout mice did not demonstrate these same improvements. In summary, IF diet in mice models suppressed inflammation after induced ICH and significantly improved both cellular markers and morphologic outcomes via the Sir3 pathway [43]. In Lee et al., IF was found to be protective against neuroinflammation and impairment in cognition associated with a high-fat diet in mice, a model commonly used to study diabetes. Mice were randomized and fed one of three diets; normal diet, high-fat diet (HFD), and high-fat diet combined with intermittent fasting. The HFD mice were fed this diet for 30 weeks, while the combined diet mice were fed HFD for 8 weeks followed by alternating day (24 h fast) IF diet for 22 weeks. The HFD mice were found to have higher levels of lipocalin-2 (LCN2) and galectin-3 (GAL3), two proteins which play inflammatory roles associated with obesity and diabetes. LCN2, for example, promotes insulin resistance and increases blood brain barrier (BBB) permeability (BBB leakage in the hippocampus is thought to be a cause of cognitive impairment in diabetes and AD), while GAL3 promotes oxidative stress and impairs learning and memory in diabetes patients. In this study, HFD mice treated with IF had downregulated LCN2 and GAL3 both in circulation and in adipose tissue macrophages. IF treated mice had reduced excess weight and improved insulin resistance brought on by HFD. IF also improved memory deficits induced by HFD, which was tested using the Morris Water Maze test. In the hippocampus, IF inhibited BBB leakage in HFD mice and reduced inflammatory markers such as tumor necrosis factor-α and interleukin-6. The authors argue that their results show that IF reversed the neuroinflammatory condition induced by HFD, suggesting that IF may be a viable treatment for HFD-generated cognitive impairment [44]. The study provides interesting evidence for the mechanism of metabolic disturbance in neurocognitive disorders, further shedding light on the relationship and providing building blocks for future studies. The ability of IF to apparently attenuate neuropathology associated with mouse models of ICH and diabetes has implications suggesting the role of an IF diet as a potential intervention for other disorders associated with neuroinflammation, including neurocognitive and mood disorders.

### 3.3 Intermittent fasting in neurocognitive disorders

As intermittent fasting has shown desirable anti-inflammatory and pro-plasticity changes in the brains of mice as discussed, the natural proceeding question is to investigate its effect on disorders of interest. Major neurocognitive disorder (MND), formerly known as dementia and characterized by “progressive and persistent” decline in cognitive function, is one of the most common yet devastatingly disabling diseases. Alzheimer’s disease makes up 70–80% of MND and is the 5^th^ leading cause of death over 65 in the U.S., with vascular disease following at 15% and other subtypes making up the rest of cases [45]. With a lack of effective and significantly disease-modifying treatments available, lifestyle modifications such as diet are of interest for their capability to impact the onset or progression of disease [46]. IF has been studied in several models of neurocognitive disease and shows potential as a lifestyle intervention for these disorders.

#### 3.3.1 Early cognitive changes with preserved activities of daily living (mild cognitive impairment) and intermittent fasting in humans

Human studies of intermittent fasting and neurocognitive disorders are lacking and provide much room for research in this area. A 3-year progressive cohort study by Ooi et al. published in 2020 studied the effect of IF on elderly individuals with mild cognitive impairment (MCI) [47]. MCI, now referred to as early cognitive changes with preserved activities of daily living (ADLs), has specific criteria in the DSM-V, but essentially covers a grey area of symptomology including cognitive decline that is out of proportion to normal aging, while not meeting the criteria for a major neurocognitive disorder, or dementia. It is relatively common, estimated at about 15–20% of the over 60 population internationally, and is a risk factor for the development of MND including AD. This makes it an important diagnostic tool and a potential target area for early intervention [48,49]. Ooi et al. studied 99 older adults with MCI over 36 months. They were randomly divided into three groups based upon IF status: regularly practicing IF (r-IF), irregularly practicing IF (i-IF), or not practicing IF controls (n-IF); regular fasting was from sunrise to sunset two days per week. At 36 months, MCI subjects in the r-IF group had better cognitive scores and reverted to better cognitive functioning compared to the i-IF or n-IF groups. Cognition was measured through the Mini mental state exam (MMSE), MOCA, Rey Auditory Verbal Learning Test, Digit span test, and digit symbol substitution test. The r-IF group also had higher antioxidant superoxide dismutase levels, reduced body weight, lowered insulin levels, reduced fasting blood glucose, less DNA damage, lower CRP levels (an inflammatory marker), and lower malondialdehyde (a marker of oxidative stress). Researchers also assessed at 36 months whether individuals with a diagnosis of MCI could be reclassified into normal or successful aging. Successful aging was defined as free from chronic disease or depression, good functional abilities, and MMSE > 22. Normal aging was no MND and MMSE between 19 and 22. Both the r-IF group and i-IF group had significantly greater numbers of participants revert to successful aging, with 24% of r-IF participants reverting to successful aging vs 14.2% in the i-IF group and only 3.7% in the n-IF controls. Results were similarly positive for normal aging. In regard to keeping the diagnosis of MCI, only 2.7% of r-IF individuals still met criteria after 36 months, compared to 22.9% of i-IF and 66.7% of n-IF controls [48]. While much is undiscovered in the realm of human research in the neurocognitive disorder arena as it relates to IF, this study provides an interesting look at the potential for IF as a lifestyle intervention and opens the door for further investigation in this area.

#### 3.3.2 Alzheimer’s disease and intermittent fasting

Alzheimer’s disease with amyloid-β and tau pathology does not occur naturally in wild-type mice but has been studied extensively using various models, including several transgenic types. Two studies looked at time-restricted feeding in transgenic AD mice. In Halagappa et al., researchers used a triple-transgenic model with mutated amyloid precursor, presenilin-1, and tau mutations to simulate AD. Mice were randomized to four groups: Non-transgenic AL, triple-transgenic transgenic AL, triple-transgenic 40% calorie restriction (CR), and triple-transgenic intermittent fasting with alternating day feeding. Half of each group were measured at 7 and half at 14 months of these protocols, then assessed using tasks and protein quantification. To examine locomotor activity and exploratory behavior, they use the Open Field Activity task. All triple-transgenic mice had reduced ambulation and distance than non-transgenic controls, but IF and CR diets lessened these deficits compared to AL feeding in the triple-transgenic mice. Similarly, AD modeling worsened performance on the Morris Swim Test, but the triple-transgenic mice fed IF or CR diets both performed better than the AL feeding triple-transgenic controls. This result held true for both male and female subgroups. However, when measuring amyloid-β and phosphorylated tau levels in the triple-transgenic AD groups, only the CR mice showed a significant lowering versus AL mice. IF did not show a significant difference in these. Because of this, authors discussed that IF likely imparts its cognitive benefits by secondary protection against adverse effects of amyloid beta and tau on synapse function (rather than direct lowering) through upregulated protein chaperones and neurotrophic factors including BDNF [50].

In another study on mouse models of AD, Whittaker et al. studied TRF without calorie restriction and looked at sleep patterns, gene expression, and amyloid-β levels [51]. Sleep-related pathology often accompanies AD with bi-directional causality. Recognition of alterations in sleep pattern may increase early detection and intervention for AD, while on the other hand treatment of sleep disturbance can improve overall disease management [52]. In this experiment, the main single-gene mutation for AD was in amyloid precursor protein-23 transgenic (APP23); Amyloid precursor protein-knock in (APP-KI) was also studied as a triple-transgenic mouse model for more aggressive AD progression. Transgenic and non-transgenic mice were randomly assigned to IF or AL feeding. The authors found that transgenic APP23 mice displayed disrupted sleep and circadian regulation, as well as disrupted hippocampal gene expression and impaired diurnal variations in transcription in the hippocampus. In the AD model mice who were fed IF/TRF diets, sleep patterns were found to be improved, particularly with sleep onset and total sleep amount. IF was also seen to modulate hippocampal transcription and inflammation pathways in the transgenic mice compared to AL transgenic mice. 86 genes associated with AD and 100 neuroinflammatory genes associated with myelination, neurotransmitter synthesis and storage, autophagy, cytokine remodeling, and immune response. In addition, IF reduced amyloid-β 40 and 42 levels and increased clearance in the brains of mice. The latter finding contrasts with Halagappa et al. discussed above, in which only calorie-reduced but not IF-diet mice had lower levels of amyloid-β. Interestingly, the APP-KI mice of this study, with more rapidly progressing disease, still showed decreased amyloid load and decreased pTau when treated with TRF. This corresponded with similar improvement in cognition on this diet. Overall, this study demonstrated that TRF initiated behavioral, cognitive, and molecular changes which improved AD pathology in mouse models [51].

#### 3.3.3 Vascular cognitive impairment and intermittent fasting

In Rajeev et al., mice were modeled with vascular cognitive impairment (VCI) to investigate potential benefits of IF on this pathology. Mice were randomized to AL or IF (16 h daily fast) feeding groups for four months. Both groups then had induction of chronic cerebral hypoperfusion to model VCI through bilateral common carotid artery stenosis, while a control group were given sham surgeries. Vascular and neuronal pathology markers were examined at 15 and 30 days, assessing for changes to leaky microvessels, BBB permeability, protein expression of tight junctions, extracellular matrix components, and white matter changes. By measuring cerebral blood flow at baseline, after stenosis surgery, and before sacrifice, it was demonstrated that hypoperfused mice treated with IF diet had more blood flow restored to the brain. IF also reduced leaky microvessel incidence compared to AL mice [53]. Microvessel leakage is a known indicator of ischemia/reperfusion injury, with resulting edema being the pathophysiological marker, due to disruptions of various contributors to the endothelial barrier during the injury. It is also a potential therapeutic target [54]. The IF-treated mice in this study had fewer leaky blood vessels, significant at 30 days post-injury. Similarly, IF-treated mice had preserved BBB permeability as opposed to AL mice, measured with Evans Blue dye concentration injected peripherally 30 minutes prior to sacrifice. BBB breakdown was reduced, and white matter damage was also less severe in IF mice. Nissl-stained neurons were examined at three regions of the hippocampus, with significantly reduced neuron degeneration in each region at both 15 and 30 days in IF versus AL mice. In terms of molecular markers, IF mice had lower levels of matrix metalloproteinase, thought to be involved in the breakdown of the extracellular matrix. Antioxidants superoxide dismutase and glutathione were both upregulated in IF mice, and a marker of oxidative stress, malondialdehyde, was reduced in IF mice compared to AL. These authors concluded that IF could be a potential therapeutic tool to attenuate VCI-related pathology [53].

### 3.4 Limitations and risks of intermittent fasting

As discussed in the previous section, intermittent fasting cannot be discussed without noting its unsuitability for certain populations. Cases where IF may be recommended against could include patients with a history of eating disorders and certain medical conditions [55]. IF has been shown to have impacts on thyroid and other hormones, although effects are not well known at this point [56]. IF is being studied and has been shown to be an effective treatment in diabetes mellitus, however extra care should be taken in this population for proper oversight and medication management [57]. A dietitian should be consulted whenever possible, especially if IF is being considered for a patient with a history of complex nutritional needs. Even considering the use of IF to avoid constant CR, care should be taken to ensure that malnutrition is avoided [58]. As with many lifestyle interventions, IF may not be an achievable treatment for some individuals. Biopsychosocial factors must be acknowledged both before and during intervention to determine appropriateness for the patient. Overall, proper oversight by medical providers using discretion is needed in patients utilizing IF. This paper should highlight the need for more human studies on IF, with particular emphasis on long-term safety and efficacy.

## 4 Discussion

This review highlights the therapeutic potential of intermittent fasting (IF) for addressing neurocognitive disorders, with evidence pointing to its effects on neuroplasticity, inflammation, and cognitive function. Preclinical studies demonstrate that IF enhances hippocampal neurogenesis and synaptic plasticity through mechanisms such as BDNF and CREB signaling, which promote neuronal growth and resilience. Additionally, IF has shown anti-inflammatory effects in animal models of Alzheimer’s disease (AD), vascular cognitive impairment (VCI), and high-fat diet-induced cognitive impairment, reinforcing its potential role in mitigating neurocognitive decline.

Despite these promising findings, the translation of preclinical results into human applications remains an area of active exploration. Human studies on IF and neurocognition are limited, with many focusing on surrogate markers such as oxidative stress or insulin sensitivity rather than direct measures of cognitive outcomes. Preliminary findings, such as those from Ooi et al., suggest that IF may improve cognitive function and metabolic health in individuals with mild cognitive impairment (MCI). However, these studies often involve small sample sizes, varied fasting protocols, and short follow-up durations, which restrict the generalizability of their conclusions. The variability in study design and the lack of standardization across fasting protocols pose additional challenges for interpreting results and comparing outcomes. Moreover, many human studies fail to address long-term safety, adherence, and the potential for IF to exacerbate comorbid conditions, such as diabetes or thyroid dysfunction. These gaps underscore the need for larger, randomized controlled trials that evaluate standardized IF protocols and assess outcomes directly related to cognitive trajectories and functional independence.

While IF holds promise as a therapeutic strategy for individuals with existing cognitive impairment, its clinical implementation must be approached cautiously. Individualized approaches that consider patient-specific nutritional needs, comorbidities, and adherence challenges are critical. Furthermore, multidisciplinary collaboration among researchers, clinicians, and dietitians will be essential to ensure safe and effective application of IF in neurocognitive care.

## 5 Conclusion

Given the growing interest in intermittent fasting as a lifestyle intervention for brain health and slowing cognitive decline, this review focuses on its effects on neuroplasticity, inflammation, neurocognitive disorders, and gives a glimpse into the use of fasting in other psychiatric disorders. As discussed, some of the decline in cognition with age and with neurocognitive disorder may be in part due to impaired glucose metabolism. The metabolic switch to ketogenesis that occurs during fasting may benefit brain health by providing alternative energy sources. Through signaling pathways such as BDNF and CREB, IF promotes hippocampal neurogenesis while increasing synaptic plasticity, allowing a possible modality for new growth to counteract loss of function. Neuroinflammation is strongly associated with cognitive disorders, such as AD and Parkinson Disease. As shown in mouse studies on intracerebral hemorrhage and high-fat diet, IF seems to play a major role in decreasing inflammation, both at the molecular and macro level. Looking further into mice modeled with major neurocognitive disorder of both vascular and AD subtypes, IF may similarly attenuate effects of brain injury and promote the brain and body’s ability to deploy natural defense systems against disease. Emerging human studies show promise, illustrating potential for IF as a tool against early neurocognitive decline. The reviewed evidence leaves many implications for future research, especially highlighting the need for more controlled studies in human populations. Studies should aim for a more comprehensive understanding of the potential risks and benefits of IF for brain health. Neurocognitive decline stands to have a widening impact on human populations as societies age and must cope with the consequences of factors, some unavoidable, which emerge along the way. A multifaceted approach of medicine and lifestyle is required to have the best chance of rivaling the diseases which threaten to take the mind and necessitates pushing the limits of science to determine what is within our control.

## Declaration of Generative AI and AI-assisted technologies in the writing process

During the preparation of this work the authors used ChatGPT version 4.0 solely in order to improve language and readability. After using this tool/service, the authors reviewed and edited the content as needed and take full responsibility for the content of the publication.

## Declaration of competing interest

The authors declare that they have no known competing financial interests or personal relationships that could have appeared to influence the work reported in this paper.
